# Water resources allocation considering water supply and demand uncertainties using newsvendor model-based framework

**DOI:** 10.1038/s41598-023-40692-7

**Published:** 2023-08-22

**Authors:** Yanhu He, Yanhui Zheng, Xiaohong Chen, Binfen Liu, Qian Tan

**Affiliations:** 1 Guangdong Provincial Key Laboratory of Water Quality Improvement and Ecological Restoration for Watersheds, Institute of Environmental and Ecological Engineering, Guangdong University of Technology, Guangzhou, 510006 People’s Republic of China; 2 Southern Marine Science and Engineering Guangdong Laboratory (Guangzhou), Guangzhou, 511458 People’s Republic of China; 3 Guangzhou Franzero Water Technology Co., Ltd., Guangzhou, 510663 People’s Republic of China; 4 State Environmental Protection Key Laboratory of Integrated Surface Water-Groundwater Pollution Control, School of Environmental Science and Engineering, Southern University of Science and Technology, Shenzhen, 518055 People’s Republic of China; 5 School of Civil Engineering, Sun Yat-Sen University, Zhuhai, 519082 People’s Republic of China

**Keywords:** Environmental sciences, Environmental social sciences, Hydrology

## Abstract

A novel newsvendor model-based framework for regional industrial water resources allocation that considers uncertainties in water supply and demand was proposed in this study. This framework generates optimal water allocation schemes while minimizing total costs. The total cost of water allocation consists of the allocated water cost, the opportunity loss for not meeting water demand, and the loss of the penalty for exceeding water demand. The uncertainties in water demand and supply are expressed by cumulative distribution functions. The optimal water allocation for each water use sector is determined by the water price, the unit loss of the penalty and opportunity loss, and the cumulative distribution functions. The model was then applied to monthly water allocation for domestic, industrial, and agricultural water use in two counties of Huizhou City, China, whose water supply mainly depends on Baipenzhu Reservoir. The water demand for each water use sector and the monthly reservoir inflow showed good fits with the uniform and P-III distributions, respectively. The water demand satisfied ratio for each water use sector was stable and increased for the optimal water allocation scheme from the newsvendor model-based framework, and the costs were lower compared with the actual water allocation scheme. The novel framework is characterized by less severe water shortages, lower costs, and greater similarity to actual water use compared with the traditional deterministic multi-objective analysis model, and demonstrates strong robustness in the advantages of lower released surplus water and higher water demand satisfied ratio. This novel framework yields the optimal water allocation for each water use sector by integrating the properties of the market (i.e., determining the opportunity loss for not meeting water demand) with the government (i.e., determining the water price and the loss of the penalty for exceeding water demand) under the strictest water resources management systems.

## Introduction

Water is a natural resource and a strategic economic resource that is essential for supporting domestic and ecological water use. Conflicts over water use can occur when water resources are scarce, such as during extreme dry events or when water use is excessive. Several national policies have exerted great pressure on the local government to improve water use efficiency and protect water quality^1^. Water use in some regions has been stable or even decreased following the implementation of these stringent water resources management regimes^2^. Regional water demand is still expected to increase due to rapid economic and population growths, and the imbalance between water supply and demand is expected to increase during certain periods, especially in rapidly developing countries and regions with scarce water resources.

Water resources allocation is essential for managing the imbalance in the spatial–temporal distribution of water resources and the mismatch between water inflow and water demand^3^, improving water use efficiency, and enhancing the resistance of the water resources system to risk^4,5^. When the total amount of water in a region is limited, determining how to optimally allocate water resources to meet societal, domestic, and economic demands while minimizing costs is of great theoretical and practical significance for supporting the sustainable development of the economy and society. Water resources allocation models have been developed using various approaches, including simulations^6^, optimization^7^, artificial intelligence approach^8^, game theory^9^, and complex adaptive systems^10^. These models have provided new insights into water resources allocation by examining the balance between economic and social water use and ecological water use^11^, and the results of this work have important practical implications. The relationship between allocated water, water inflows, and water demands during specific periods have not been further studied in these water resources allocation models; the cost of water allocation is still insufficiently examined by previous water resources allocation studies^12,13^. For example, penalties for exceeding total constrained water use have not been properly quantified or incorporated as a hard constraint in an optimization model, and this impedes the implementation of stringent water resources management regimes.

The achievement of rationality in cost allocation and the planning of cost allocations when water demands change in time remain the challenging operational research problems^14^. In previous studies, on one hand, limited type of water use cost was considered in the cost-minimization model. The type of water use cost usually includes the operation cost^15,16^, total system cost^17^, supply cost^18,19^, which covers the costs related to the exploitation, utilization and protection of water resources in terms of water price. In fact, water resources are an important basis for maintaining the health of life, industrial and agricultural productions and ecological environment whereas water resources are limited in a certain range of time and space, which makes it necessary to rationally allocate water resources in various regions, water use sectors and time periods, so as to maximize the economic, social and ecological benefits of water utilization. If the allocated water is greater than the actual water demand, there would be waste of water use and it should be punished, particularly in some countries or regions with strict water management system^20,21^; in contrast, if the allocated water is less than the actual water demand, there will be economic loss due to water shortage, that is the opportunity loss. Besides the above costs, the total cost of water use should also contain the loss of the penalty for exceeding water demand and the opportunity loss for not meeting water demand, which have not been properly considered in the previous studies. As the two core elements of water resources allocation, both available water resources and water demand show uncertainties aggravated by climate change and intensive human activities, while previous studies on water resources allocation involving water use cost have not fully taken into account the uncertainty of available water resources and water demand. Several studies considered the uncertainty of hydrologic, water demand and economic factors when optimizing water allocation, the penalty loss and the opportunity loss have not been reflected in the economic optimization of objective functions^22–24^. It is necessary to integrate the total water use cost (the allocated water cost, the penalty loss and the opportunity loss) with both uncertainties of available water resources and water demand in the water allocation model, which has, to our knowledge, not yet been addressed.

In recent years, several strict water management systems have been established to guarantee water supply security via preventing waste of water use and improving water use efficiency, in particular to the fast-developing countries like China. For instance, in China, the Chinese central government launched the most stringent water management system as the annual “No. 1 Document” in January 2011^25^, and the red line for the constrained total water use is one of the “Three Red Lines” (i.e., the other two red lines are : (1) the red line for the water use efficiency, and (2) the red line for the pollutants released into water function areas), how to determine the constrained total water use for each region is key to the implement of the three red lines and the effective water resources management. It is urgent to develop a water resources allocation model to work out the optimal allocated water that minimizes the total water use cost (i.e., the sum of the allocated water cost, the penalty loss and the opportunity loss), and the optimal allocated water provides the basis for the constrained total water use for each region.

Newsvendor models are powerful tools for solving uncertain decision-making problems^26^. The newsvendor problem is one of the basic problems of random storage management, and it has been widely applied in many fields such as the ordering and storage of goods^27,28^, product distribution^29^, and the retail and service industry in economic management^30^. Newsvendor models have become increasingly popular in recent years as the circulation cycle of goods has shortened. Extensive research on newsvendor models has generated several important findings, including different objective functions and utility functions^31^ and different supply price strategies^32^. Current research on newsvendor models has mainly focused on the framework of probability theory (i.e., the uncertainty in supply and demand represented by a probability distribution), which aims to maximize expected income or achieve target income. Unforeseeable actual demand is the focus of the newsvendor model and is usually represented by a fuzzy membership function and probability distribution^33,34^. In the classical newsvendor model, a newsvendor buys newspapers from the newspaper office every morning for retail and returns unsold ones in the evening. The number of newspapers sold each day can be estimated, but the demand cannot be precisely predicted. Sometimes all the newspapers are sold, and sometimes there is a surplus of newspapers; if the newsvendor buys more newspapers in a day, he loses money for the return cost; if not enough newspapers are sold, he makes less money for the opportunity loss cost. The newsvendor has to decide the quantity of newspapers to buy every morning, minimizing the total cost (i.e., the sum of newspaper cost, the return loss and the opportunity loss). An optimization model of the quantity of purchased newspapers needs to be constructed to minimize the total cost. This is similar to the optimal water allocation under the most stringent water management system, in which the three types of cost and both the uncertainties in available water resources and water demands should be considered. The newsvendor problem is one of the most basic problems of random storage management, and it has been widely applied in many fields of economic management^35–37^, while it is barely applied in water resources management.

He et al.^38^ applied the Newsvendor model to allocate regional water resources under the constraint of total water-use quota and provided optimal water allocation schemes for each sector and water governor in water resources management, however, uncertainties in water demand and supply are not considered and the total water use cost cannot be properly addressed. The impacts of changes in water price, the penalty for exceeding water demand and the opportunity loss for not meeting water demand on the optimal water allocation aren’t investigated. The novelty of the current study is to propose a newsvendor model-based water resources allocation framework. This framework considers the uncertainties both in water inflows and demands, minimizes the total cost of allocated water and penalties for allocated water exceeding or failing to satisfy future water demand in the water resources allocation model. This newsvendor model improves the optimization of water resources allocation for the regional industrial water use sectors and was applied to optimize the monthly water allocation for domestic, industrial, and agricultural water use in two counties of Huizhou City, China to evaluate the efficiency of the proposed framework. Given that the original newsvendor model only considers uncertainty in demand, uncertainty in supply is also considered in the improved newsvendor model. Both the uncertainties in water inflow and demand are considered in the optimized water allocation scheme based on the improved newsvendor model (Abbreviated as WAINM, i.e., the first letter of “Water Allocation Improved Newsvendor Model”); uncertainties in both water inflows and demands are represented by probabilistic distributions. In addition to the allocated water cost, penalty losses for allocated water exceeding future water demand and opportunity losses for allocated water failing to satisfy future water demand are also quantified and incorporated into the objective function. The WAINM is developed to balance the relationship between uncertain water inflow and demand as well as allocated water for optimized water allocation schemes while minimizing total costs. A two-stage heuristic algorithm is proposed to solve the WAINM and obtain the optimal water allocation schemes.

The specific objectives of the current study are to (i) consider uncertainties in water demand and supply using cumulative distribution functions; (ii) construct the WAINM and evaluate the its efficiency, and (iii) reveal the impacts of water price, the penalty for exceeding water demand and the opportunity loss for not meeting water demand on the optimal water allocation. The results of this study will aid regional water allocation under stringent water resources management systems.

## Proposed framework

**Figure 1:**
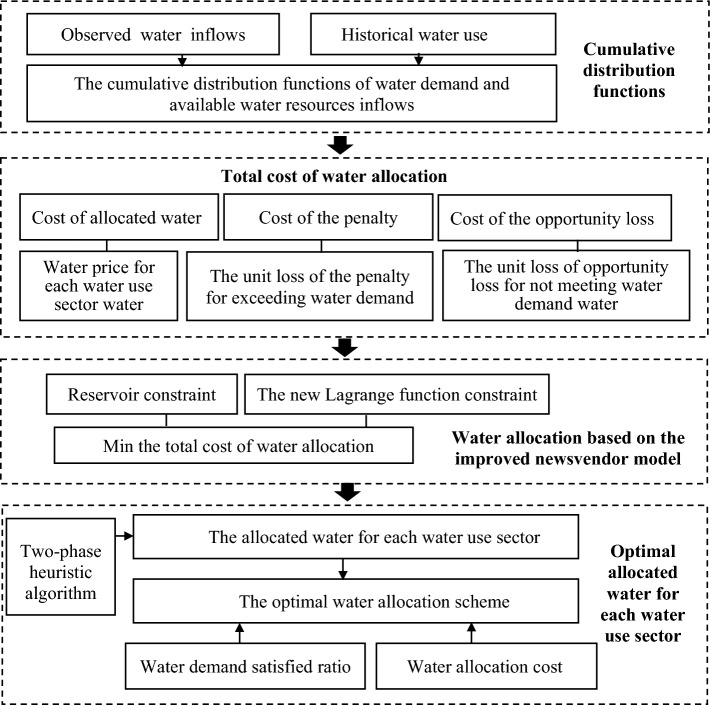
Flowchart of the framework.

In the proposed framework (Fig. 1), several possibility distribution functions are used to simulate the uncertainties in water demand and available water resources, and the cumulative distribution functions (CDFs) of water demand and available water resources are obtained. Next, the water allocation model (based on the newsvendor model) is constructed, and the solution method is chosen. The optimal water allocation scheme with the lowest total cost is determined.

### Water allocation based on the newsvendor model

#### The classical newsvendor model

In the classical newsvendor model, the cost of each newspaper is $c$. Sometimes all the newspapers are sold, and sometimes there is a surplus of newspapers; if the newsvendor buys more newspapers in a day, he loses money at the return cost of $h$ for each newspaper; if not enough newspapers are sold, he makes less money at the opportunity loss cost of $v$. An optimization model of the quantity of newspapers to purchase needs to be constructed to minimize the total cost. Newsvendor models are powerful tools for solving problems associated with uncertain decision making. The newsvendor problem is one of the most basic problems of random storage management, and it has been widely applied in many fields of economic management, such as goods ordering and storage and product distribution.

#### Newsvendor model-based framework for water allocation that considers the uncertainties in water supply and demand

1. Basic idea

Like a newsvendor selling newspapers (i.e., water allocation), when the unit purchasing cost (i.e., $c$) of newspaper (i.e., the purchase cost of a unit water resource or water price), the unit loss (i.e., $h$) of the over-purchased part (i.e., the penalty for exceeding water demand), and the unit loss (i.e., $v$) of the under-purchased part (i.e., the opportunity loss for not meeting water demand) are known, and the exact demand (i.e., water demand during a period) is unknown, the number of newspapers the newsvendor should purchase every day (i.e., how much water should be allocated) needs to be determined to minimize the total cost (i.e., the total cost of water allocation). One of the basic assumptions of the newsvendor model is that the demand for newspapers is uncertain, which corresponds to the uncertainty in the water demand of each water use sector in the water resources allocation model. There is no limit to the quantity of goods in the classical newsvendor model. The available water resources are limited and uncertain under changing environments; available water resources are limited by the engineering capacity. The classical newsvendor model needs to be improved to develop a water resources allocation model that considers the uncertainties in both water supply and demand.

The main advantages gained by the analogy of water allocation to the newsvendor problem are as follows: (1) the allocated water cost as well as the penalty and opportunity loss can be properly considered when allocating water resources and determining the constrained total water use, which fits well with the most stringent water management system; (2) the impacts of changes in water price, the penalty for exceeding water demand and the opportunity loss for not meeting water demand on the optimal water allocation can be quantified via the newsvendor model-based water resources allocation framework, which aids water governor for effective water resources management.

(2) Improved newsvendor-based framework for water allocation that considers the uncertainties in water supply and demand

Water resources allocation involves reasonably allocating limited water resources among different water use sectors and ensuring the efficient utilization of water resources. If a region is divided into $i$ ($i=\mathrm{1,2},3,\ldots , I$) calculation units, there are $j$ ($j=\mathrm{1,2},3,\ldots , J$) water use sectors for each calculation unit. The objective function based on the newsvendor model is determined to minimize the total cost of water allocation. The cost function of the ${j}_{th}$ water use sector in the ${i}_{th}$ calculation unit is as follows:

1 $$\mathrm{C}\left({x}_{i,j}^{t}\right)={C}_{o}\left({x}_{i,j}^{t}\right)+{C}_{u}\left({x}_{i,j}^{t}\right)+{C}_{p}({x}_{i,j}^{t})$$

The expected cost functions are as follows:

2 $$E[{C}_{o}\left({x}_{i,j}^{t}\right)]=h\cdot \underset{0}{\overset{\infty }{\int }}\mathrm{g}\left({R}^{\mathrm{t}}\right)\underset{0}{\overset{{x}_{i,j}^{t}}{\int }}\left({x}_{i,j}^{t}-{D}_{i,j}^{t}\right)\cdot {f}_{i,j}\left({D}_{i,j}^{t}\right)\cdot \mathrm{d}{D}_{i,j}^{t}\cdot \mathrm{d}{R}^{t}$$

3 $$E[{C}_{u}\left({x}_{i,j}^{t}\right)]=v\cdot \underset{0}{\overset{\infty }{\int }}\mathrm{g}\left({R}^{t}\right)\underset{{x}_{i,j}^{t}}{\overset{\infty }{\int }}\left({D}_{i,j}^{t}-{x}_{i,j}^{t}\right)\cdot {f}_{i,j}\left({D}_{i,j}\right)\cdot \mathrm{d}{D}_{i,j}^{t}\cdot \mathrm{d}{R}^{t}$$

4 $$E[{C}_{p}\left({x}_{i,j}^{t}\right)]=c\cdot {x}_{i,j}^{t}$$

where, in time period $t$, ${D}_{i,j}^{t}$ represents the water demand; ${x}_{i,j}^{t}$ represents the allocated water resources; ${R}^{t}$ and $\mathrm{g}\left({R}^{t}\right)$ represent the runoff and its density function, respectively; and ${C}_{o}$, ${C}_{u}$, and ${C}_{p}$ represent the expected loss of the penalty for exceeding water demand, the expected opportunity loss for not meeting water demand, and the expected cost of allocated water, respectively.

$\mathrm{C}\left({x}_{i,j}^{t}\right)$ is a concave function that needs to be minimized to optimize the allocation of water. The upper and lower bounds of runoff and water demand need to be compared to define $\mathrm{C}\left({x}_{i,j}^{t}\right)$.

5 $${D}_{i,jmin}^{t}<{D}_{i,j}^{t}<{D}_{i,jmax}^{t}$$

6 $${R}_{min}^{t}<{\mathrm{R}}^{t}<{R}_{max}^{t}$$

If $${R}_{max}^{t}<{D}_{i,jmax}^{t}$$,

7 $$E[{C}_{o}\left({x}_{i,j}^{t}\right)]=h\cdot \underset{{R}_{min}^{t}}{\overset{{R}_{max}^{t}}{\int }}\mathrm{g}\left(\mathrm{R}\right)\underset{0}{\overset{{x}_{i,j}^{t}}{\int }}\left({x}_{i,j}^{t}-{D}_{i,j}^{t}\right)\cdot {f}_{i,j}\left({D}_{i,j}^{t}\right)\cdot \mathrm{d}{D}_{i,j}^{t}\cdot {\mathrm{dR}}^{t}$$

8 $$E[{C}_{u}\left({x}_{i,j}^{t}\right)]=v\cdot \underset{{R}_{min}^{t}}{\overset{{R}_{max}^{t}}{\int }}\mathrm{g}\left(\mathrm{R}\right)\underset{{x}_{i,j}^{t}}{\overset{{D}_{i,jmax}^{t}}{\int }}\left({D}_{i,j}^{t}-{x}_{i,j}^{t}\right)\cdot {f}_{i,j}\left({D}_{i,j}^{t}\right)\cdot \mathrm{d}{D}_{i,j}^{t}\cdot {\mathrm{dR}}^{t}$$

9 $${F}_{i,j}\left({{x}_{i,j}^{t}}^{*}\right)=\frac{v-c}{h+v}$$

where ${{x}_{i,j}^{t}}^{*}$ represents the optimal allocated water, and ${F}_{i,j}()$ represents the CDF of the water demand.

If $${R}_{max}^{t}>{D}_{i,jmax}^{t}$$,

10 $$E[{C}_{o}\left({x}_{i,j}^{t}\right)]=h\cdot \underset{{R}_{min}^{t}}{\overset{{D}_{i,jmax}^{t}}{\int }}\mathrm{g}\left({\mathrm{R}}^{t}\right)\underset{0}{\overset{{x}_{i,j}^{t}}{\int }}\left({x}_{i,j}^{t}-{D}_{i,j}^{t}\right)\cdot {f}_{i,j}\left({D}_{i,j}^{t}\right)\cdot \mathrm{d}{D}_{i,j}^{t}\cdot {\mathrm{dR}}^{t}+h\cdot \underset{{D}_{i,jmax}^{t}}{\overset{{R}_{max}^{t}}{\int }}\mathrm{g}\left({\mathrm{R}}^{t}\right)\underset{0}{\overset{{D}_{i,jmax}^{t}}{\int }}\left({x}_{i,j}^{t}-{D}_{i,j}^{t}\right)\cdot {f}_{i,j}\left({D}_{i,j}^{t}\right)\cdot \mathrm{d}{D}_{i,j}^{t}\cdot {\mathrm{dR}}^{t}$$

11 $$E[{C}_{u}\left({x}_{i,j}^{t}\right)]=v\cdot \underset{{R}_{min}^{t}}{\overset{{D}_{i,jmax}^{t}}{\int }}\mathrm{g}\left({\mathrm{R}}^{t}\right)\underset{{x}_{i,j}^{t}}{\overset{{D}_{i,jmax}^{t}}{\int }}\left({D}_{i,j}^{t}-{x}_{i,j}^{t}\right)\cdot {f}_{i,j}\left({D}_{i,j}^{t}\right)\cdot \mathrm{d}{D}_{i,j}^{t}\cdot {\mathrm{dR}}^{t}$$

12 $${F}_{i,j}\left({{x}_{i,j}^{t}}^{*}\right)=1-\frac{h+c}{(h+v)G({D}_{i,jmax}^{t})}$$

where ${{x}_{i,j}^{t}}^{*}$ represents the optimal allocated water, and the derivation procedure is provided in D1 in the supplementary information file.

In contrast to the unlimited order quantity in the classical Newsvendor model, available water resources in water allocation are constrained by the reservoir capacity. That is, the total amount of allocated water is limited by the discharge of the reservoir, and the discharge is also restricted by the design water level of the reservoir. The constraints are as follows:

1. Reservoir capacity constraint
  13 $${V}_{min}\le {V}^{t}\le {V}_{max}$$
2. Water balance constraint
  14 $${V}^{t}={V}^{t-1}+{r}^{t}-{Q}^{t}$$
3. Reservoir discharge constraint
  15 $$\sum_{i=1}^{I}\sum_{j=1}^{J}{x}_{i,j}^{t}\le {Q}^{t}$$

Equation (16) can be obtained from Eqs. (13) and (14):

16 $${V}^{t-1}+{r}^{t}-{V}_{max}\le {Q}^{t}\le {V}^{t-1}+{r}^{t}-{V}_{min}$$

The maximum reservoir discharge ${{Q}_{max}^{t}=V}^{t-1}+{r}^{t}-{V}_{min}$

where ${V}^{t},$ ${r}^{t}$, and ${Q}^{t}$ represent reservoir capacity, inflow, and outflow in time period $t$, respectively.

The optimal allocated water ${{x}_{i,j}^{t}}^{*}$ can be obtained according to the above expected cost function and constraints. If ${V}^{t}<{V}_{min}$,

17 $$\sum_{i=1}^{I}\sum_{j=1}^{J}{{x}_{i,j}^{t}}^{*}>{Q}_{max}^{t}={V}^{t-1}+{r}^{t}-{V}_{min}$$

then the total allocated water exceeds the maximum discharge of the reservoir; ${{x}_{i,j}^{t}}^{*}$ is not the optimal allocated water and requires modification. In the current study, a two-phase heuristic algorithm was introduced to obtain the optimal allocated water ${{x}_{i,j}^{t}}^{*}$. In this method, a new Lagrange function is constructed by linking the expected cost function with the constraint of available water resources through Lagrange parameters (e.g., $\lambda$), and then the newsvendor model of water resources allocation under limited available water resources is solved by solving the new Lagrange function.

The new Lagrange function is as follows:

18 $$\mathrm{L}({x}_{i,j}^{t},\lambda )=\sum_{i=1}^{I}\sum_{j=1}^{J}[{C}_{o}\left({x}_{i,j}^{t}\right)+{C}_{u}\left({x}_{i,j}^{t}\right)+{C}_{p}\left({x}_{i,j}^{t}\right)]+\lambda (\sum_{i=1}^{I}\sum_{j=1}^{J}{x}_{i,j}^{t}-{Q}_{max}^{t})$$

where $\lambda$ represents the Lagrange parameter. The optimal allocated water ${{x}_{i,j}^{t}}^{*}$ can be obtained as follows:

19 $${\text{If}}\,R_{max} < D_{i,jmax} ,F_{i,j} \left( {x_{i,j}^{t} \,^{*} } \right) = \frac{v - c - \lambda }{{h + v}}$$

20 $${\text{If}}\,R_{max} > D_{i,jmax} ,F_{i,j} \left( {x_{i,j}^{t} \,^{*} } \right) = 1 - \frac{h + c - \lambda }{{\left( {h + v} \right)G\left( {D_{i,jmax}^{t} } \right)}}$$

where ${{x}_{i,j}^{t}}^{*}$ represents the optimal allocated water, which can be represented by $\lambda$. An optimal value of $\lambda$ is calculated to obtain the optimal allocated water ${{x}_{i,j}^{t}}^{*}$. ${{x}_{i,j}^{t}}^{*}$ is determined by the water price ($c$), the unit loss of the penalty for exceeding water demand ($h$), and the unit loss ($v$) of opportunity loss for not meeting the water demand of the ${j}_{th}$ water use sector in the ${i}_{th}$ region. The CDFs of water use and reservoir inflow also affect the optimal allocated water.

The detailed derivation procedure is provided in D2 in the supplementary information file.

### Uniform and P-III distributions

The uniform distribution has been a useful tool for describing the distribution of some water-related elements such as irrigated water depth^39^ and daily streamflow^40^. Given the rapid development of the agricultural industry and population growth, data for the first three years of the forecasted years were used for the historical data analysis. We assumed that agricultural, industrial, and urban water demand fit the uniform distribution^41,42^, and the highest value in the previous three years serves as the upper bound and the lowest value serves as the lower bound. That is,

21 $${f}_{i,j}\left(x\right)=\frac{1}{{D}_{max}-{D}_{min}},x\in [{D}_{min},{D}_{max}]$$

22 $${\mathrm{F}}_{i,j}\left(x\right)=\frac{x-{D}_{min}}{{D}_{max}-{D}_{min}},x\in \left[{D}_{min},{D}_{max}\right],{\mathrm{F}}_{i,j}\left(x\right)=1\mathrm{ if x}\ge {D}_{max}$$

P-III is the most commonly used probability distribution for calculating the frequency of the main hydrological elements (e.g., rainfall, runoff) in China in recent decades^43^. The P-III distribution function is as follows:

23 $$G\left(x\right)=\frac{1}{\alpha \Gamma (\gamma )}{\int }_{0}^{+\infty }({\frac{x-\beta }{\alpha })}^{\gamma -1}exp(-(\frac{x-\beta }{\alpha }))dx$$

where $\gamma$ represents the unknown shape parameter and $\Gamma (\gamma )$ is a gamma function.

24 $${x}_{p}=\overline{x}(1+{{C}_{v}}^{2}\times \frac{{C}_{s}}{{C}_{v}}\times \frac{Gammainv\left(1-p,\alpha ,\beta \right)}{2}-2\frac{{C}_{v}}{{C}_{s}})$$

Equation (24) can be obtained by the standardized transformation of Eq. (23). P-III is used to fit the historical monthly inflow series, and the design monthly inflow can be calculated according to the design frequency.

25 $$\mathrm{g}\left(\mathrm{R}\right)=\frac{{\beta }^{\gamma }}{\Gamma \left(\gamma \right)}{(R-{a}_{0})}^{\gamma -1}{exp}^{-\beta (x-{a}_{0})}$$

26 $$G\left(R\right)=\frac{{\beta }^{\gamma }}{\Gamma (\gamma )}{\int }_{R}^{\infty }{(R-{a}_{0})}^{\gamma -1}{exp}^{-\beta (x-{a}_{0})}dR$$

## Application of the water allocation model for Baipenzhu Reservoir, Dongjiang River Basin, China

### Basic information

**Figure 2:**
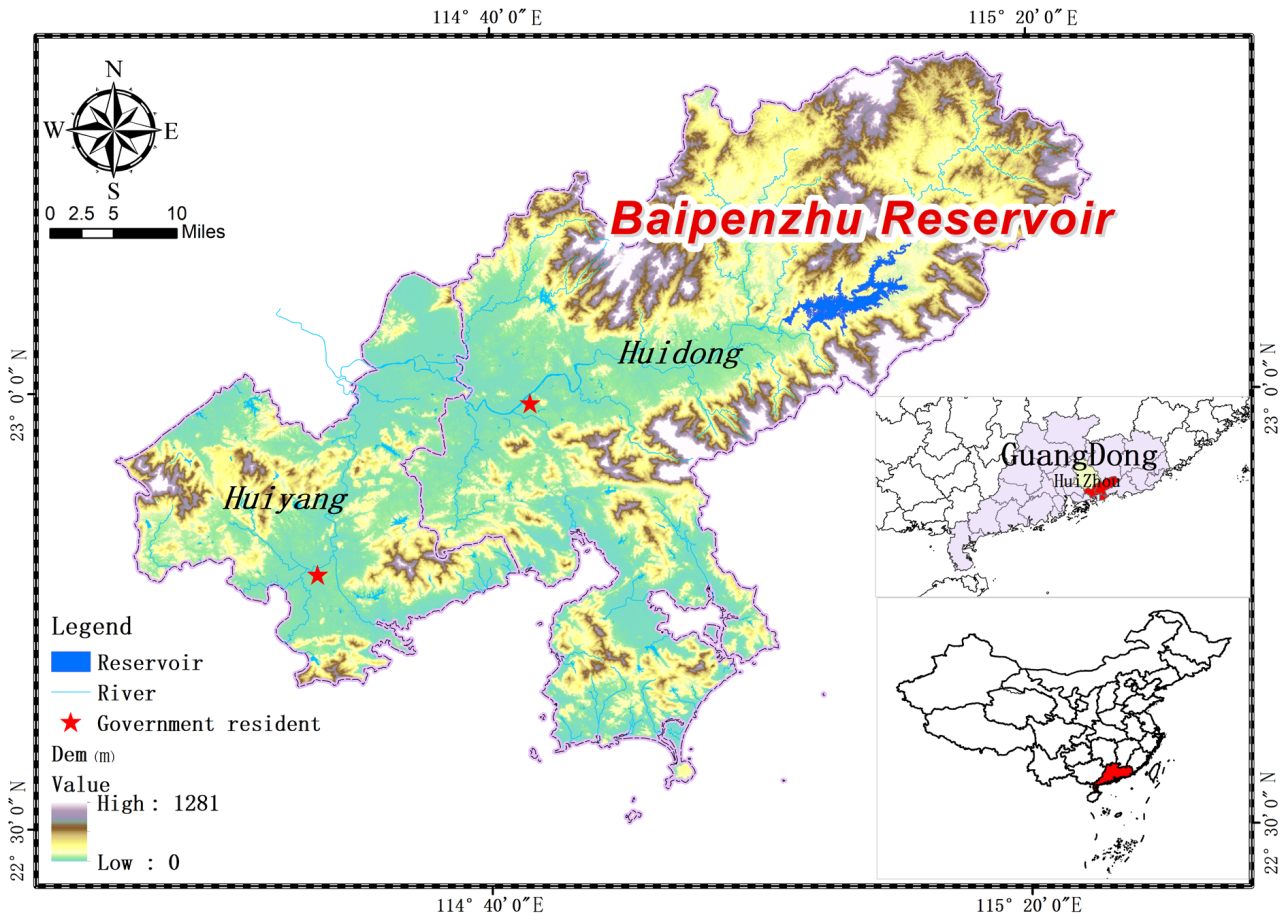
Location of the study region (this figure was generated using ArcGIS 10.4 http://appsforms.esri.com/products/download/index.cfm?fuseaction=download.main&downloadid=1932).

Baipenzhu Reservoir is located in the upper stream of the Xizhi River (Fig. 2); it was constructed in April 1958 and has a drainage area of 856 km^2^ and total reservoir capacity of 1.22 billion m^3^. Baipenzhu Reservoir is a large reservoir that functions in flood control, irrigation, power generation, and navigation. It also serves as one of the main water sources of Huidong and Huiyang, which are the two counties of Huizhou City, Guangdong Province. The two counties have exhibited stable industrial water use in recent decades; nevertheless, there is an urgent need to develop a model to optimize the allocation of the scarce water resources of Baipenzhu Reservoir among the different uses of industrial water in the two counties.

### Data

The uniform distribution was applied to analyze the uncertainties in the water demand of Huidong and Huiyang in terms of the design frequency, according to the time series of historical water use data collected from the Guangdong Water Resources Bulletin and Huizhou Statistical Yearbook (2002–2011). The P-III distribution was applied to simulate the monthly inflow of Baipenzhu Reservoir under different frequencies according to the observed inflow of the reservoir from 1985 to 2011, the data of which were obtained from the Guangdong Hydrology Bureau. The water price of each water use sector and the unit loss of the penalty for exceeding water demand are shown in Table S1. Table S1 also shows the unit loss of opportunity loss for not meeting water demand.

## Results and discussion

### Water demand and supply inputs

1. The design monthly inflow of Baipenzhu Reservoir

The frequency curves of the inflow of Baipenzhu Reservoir from Jan to Dec are shown in Fig. S1, and the correlation coefficient of each month is shown in Table S2. The degree of fit of the P-III frequency curve was very high, and the minimum correlation coefficient was as high as 0.9. The P-III frequency curve could be used to fit the monthly inflow distribution of Baipenzhu Reservoir.

(2) Industrial water demand

Table 1 shows the upper and lower bounds of the agricultural, industrial, and domestic water demand from 2005 to 2011 in Huidong and Huiyang. Industrial and domestic water consumptions were assumed to have an even intra-annual distribution (Table S3), and the intra-annual distribution of agricultural water consumption was deduced from the average water use from 2002 to 2011. Since the development of agriculture is stable, the average errors of the prediction of agricultural water demand are small, with a maximum of 8.01% and minimum of 2.07% (Table 1). Due to the rapid development of the industrial economy and population growth in the study area^44^, the prediction errors of industrial and domestic water demand were larger; the maximum average prediction error of the industrial and domestic water demand was 34.52% and 17.38%, respectively. The prediction error decreased gradually with time, and the minimum average prediction error of industrial and domestic water demand was -1.07% and -0.94%, respectively. The assumption that the water demand fits the uniform distribution is reasonable; the water demand can be predicted by the probability distribution function deduced from historical water use data.

**Table 1:** The upper and lower bounds of predicted and historical water use (million m^3^).

Year	2005	2006	2007	2008	2009	2010	2011
**Huidong**							
Agriculture							
Observed	376.2	370.3	363.3	342.3	345.0	330.8	334.0
Upper bound	409.8	397.7	376.2	376.2	370.3	363.3	345.0
Average	387.6	381.5	370.8	369.7	356.3	352.8	337.9
Lower bound	365.4	365.4	365.4	363.3	342.3	342.3	330.8
Average error (%)	3.04	3.05	2.07	8.01	3.27	6.66	1.17
Industrial							
Observed	112.8	109.6	121.4	144.3	146.7	155.0	151.3
Upper bound	80.6	112.8	112.8	121.4	144.3	146.7	155.0
Average	73.9	90.0	96.7	115.5	127.0	134.1	149.7
Lower bound	67.2	67.2	80.6	109.6	109.6	121.4	144.3
Average error (%)	− 34.52	− 17.89	− 20.35	− 19.93	− 13.46	− 13.52	− 1.07
Domestic							
Observed	86.0	94.3	97.3	104.3	94.3	99.7	100.2
Upper bound	80.9	86.0	94.3	97.3	104.3	104.3	104.3
Average	75.4	77.9	82.1	91.6	99.3	99.3	99.3
Lower bound	69.9	69.9	69.9	86.0	94.3	94.3	94.3
Average error (%)	− 12.35	− 17.38	− 15.61	− 12.11	5.27	− 0.40	− 0.94
**Huiyang**							
Agriculture							
Observed	134.5	132.4	129.9	122.4	123.3	118.2	119.4
Upper bound	146.5	142.2	134.5	134.5	132.4	129.9	123.3
Average	138.6	136.4	132.5	132.2	127.4	126.1	120.8
Lower bound	130.6	130.6	130.6	129.9	122.4	122.4	118.2
Average error (%)	3.04	3.05	2.07	8.01	3.27	6.66	1.17
Industrial							
Observed	40.3	39.2	43.4	51.6	52.4	55.4	54.1
Upper bound	28.8	40.3	40.3	43.4	51.6	52.4	55.4
Average	26.4	32.2	34.6	41.3	45.4	47.9	53.5
Lower bound	24.0	24.0	28.8	39.2	39.2	43.4	51.6
Average error (%)	− 34.53	− 17.89	− 20.35	− 19.93	− 13.46	− 13.52	− 1.07
Domestic							
Observed	30.7	33.7	34.8	37.3	33.7	35.6	35.8
Upper bound	28.9	30.7	33.7	34.8	37.3	37.3	37.3
Average	26.9	27.9	29.3	32.8	35.5	35.5	35.5
Lower bound	25.0	25.0	25.0	30.7	33.7	33.7	33.7
Average error (%)	− 12.37	− 17.38	− 15.61	− 12.11	5.27	− 0.40	− 0.94

### Water allocation based on the improved newsvendor model

#### Water demand satisfied ratio

**Figure 3:**
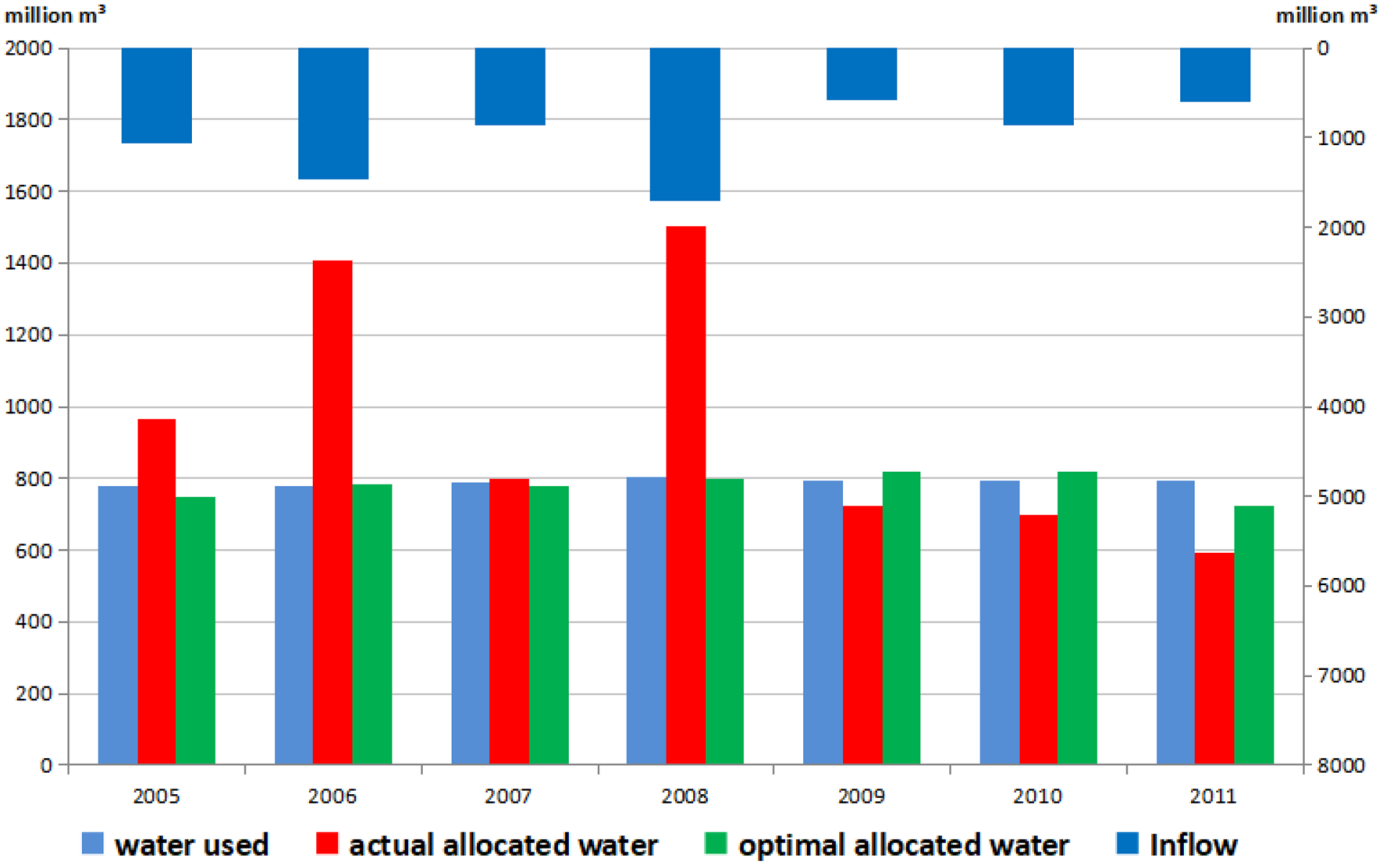
Comparison of water use and actual and optimal allocated water (Unit: million m^3^).

**Figure 4:**
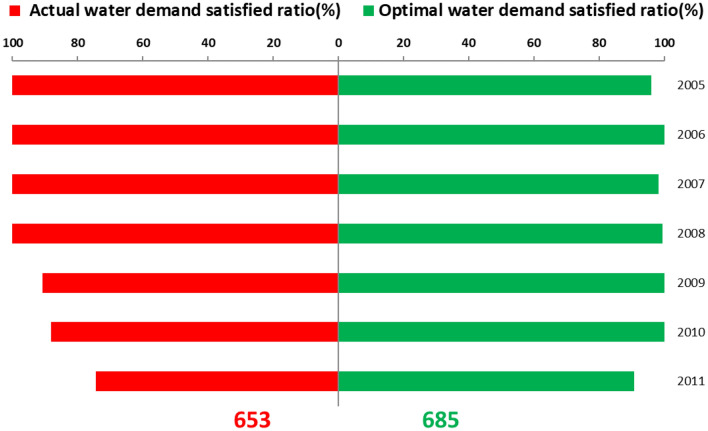
Comparison of the overall water demand satisfied ratio under actual and optimal water allocation.

Figure 3 shows actual water use, actual allocated water, and optimal allocated water by the WAINM and the inflow of Baipenzhu Reservoir from 2005 to 2011. The actual allocated water is greater than the optimal allocated water when the reservoir inflow is higher (such as for 2006 and 2008) and more surplus water is released from the reservoir (Fig. 3). Basically, higher reservoir inflow corresponds to a higher water demand satisfied ratio (Table 2) (e.g., 100% from 2005 to 2008). The water demand satisfied ratio is lower when there are fewer inflows (e.g., from 2009 to 2011). A greater release of surplus water from the reservoir in actual allocation causes the available water resources in the reservoir to be insufficient and lowers the water demand satisfied ratio during the latter period (Table 2). Although there was some variation in water inflow, both the optimal allocated water and actual water use were stable, and the optimal allocated water of each year was almost equal to the actual water use, ensuring that the water satisfied ratio remains high and stable in optimal allocation (Fig. 3). This is superior to actual allocated water, which shows consistent decrease and greater variation. This poses a major challenge for water supply security. One percent of the water demand satisfied ratio corresponds to one score; the number of scores for actual allocated water was 653, which is lower than that of optimal allocated water with 685 scores (Fig. 4), demonstrating the greater effectiveness of the WAINM.

**Table 2:** Comparison of the water demand satisfied ratio and water surplus under actual and optimal water allocation.

Item	Year	2005	2006	2007	2008	2009	2010	2011
Water demand satisfied ratio (%)	Actual allocation	100	100	100	100	91	88	74
	Optimal allocation	96	100	98	99	100	100	91
Water surplus (million m^3^)	Actual allocation	185	630	10	701	0	0	0
	Optimal allocation	23	21	12	39	32	38	3

Tables 3, 4 and Table S4 show the actual water use, optimal allocated water and actual allocated water from 2005 to 2011 for each water use sector, respectively. During this period, the agricultural, industrial and domestic water use respectively shows significant decrease, significant increase and fluctuating increase (Table 3). This is mainly due to the improved water use efficiency and the adoption of water saving technologies under the strictest water resources management systems. In the optimal water allocation, the optimal allocated water for each water use sector is determined according to the water price, the unit loss of the penalty for exceeding water demand, and the unit loss of opportunity loss for not meeting the water demand, as well as the maximum monthly water demand and water inflow, and shows similar change trend to that of the actual water use (Table 4), whereas in the actual water allocation (Table S4), the actual allocated water for each water use sector shows greater variation and doesn’t match the actual water use well compared to the optimal allocated water. In the actual water allocation, higher reservoir inflow permitted the allocation of water to each water use sector to meet water demand from 2005 to 2008; a higher water demand satisfied ratio was achieved during this period, and more surplus water was released from the reservoir (Table S5). This also resulted in a lower water demand satisfied ratio from 2009 to 2011 given that reservoir inflow was lower during this period, and more surplus water was released from the reservoir during the earlier period. The overall increasing and stable water demand satisfied ratio in the optimal water allocation scheme demonstrated the superiority of the WAINM compared with the actual water allocation.

**Table 3:** Actual water use from 2005 to 2011 (million m^3^).

Year	Huidong			Huiyang			The whole basin			
	Agricultural	Industrial	Domestic	Agricultural	Industrial	Domestic	Agricultural	Industrial	Domestic	Total
2005	376.2	112.9	86.0	134.5	40.3	30.7	510.6	153.2	116.7	780.5
2006	370.3	109.6	94.3	132.4	39.2	33.7	502.6	148.8	128.0	779.4
2007	363.3	121.5	97.3	129.9	43.4	34.8	493.1	164.9	132.0	790.0
2008	342.3	144.3	104.3	122.4	51.6	37.3	464.7	195.9	141.5	802.1
2009	345.0	146.7	94.3	123.3	52.4	33.7	468.3	199.2	128.0	795.5
2010	330.8	155.0	99.7	118.2	55.4	35.6	449.0	210.5	135.3	794.8
2011	334.0	151.3	100.2	119.4	54.1	35.8	453.4	205.4	136.1	794.8

**Table 4:** Optimal allocated water by the Water allocation based on the Improved Newsvendor Model (million m^3^).

Year	Huidong			Huiyang			The whole basin			
	Agricultural	Industrial	Domestic	Agricultural	Industrial	Domestic	Agricultural	Industrial	Domestic	Total
2005	392.7	78.9	79.9	141.2	27.9	28.4	533.9	106.8	108.3	749.0
2006	385.2	107.2	84.6	138.3	37.1	30.0	523.6	144.3	114.6	782.4
2007	372.0	108.8	92.2	133.2	38.1	32.6	505.2	146.9	124.8	776.9
2008	371.2	120.0	96.3	132.9	42.6	34.3	504.1	162.6	130.6	797.2
2009	359.5	140.0	103.4	129.0	49.1	36.8	488.5	189.1	140.2	817.8
2010	355.2	143.6	103.4	127.4	50.7	36.8	482.6	194.2	140.2	817.0
2011	275.6	152.1	102.4	101.7	53.9	36.3	377.3	206.0	138.8	722.1

#### Water allocation cost

**Figure 5:**
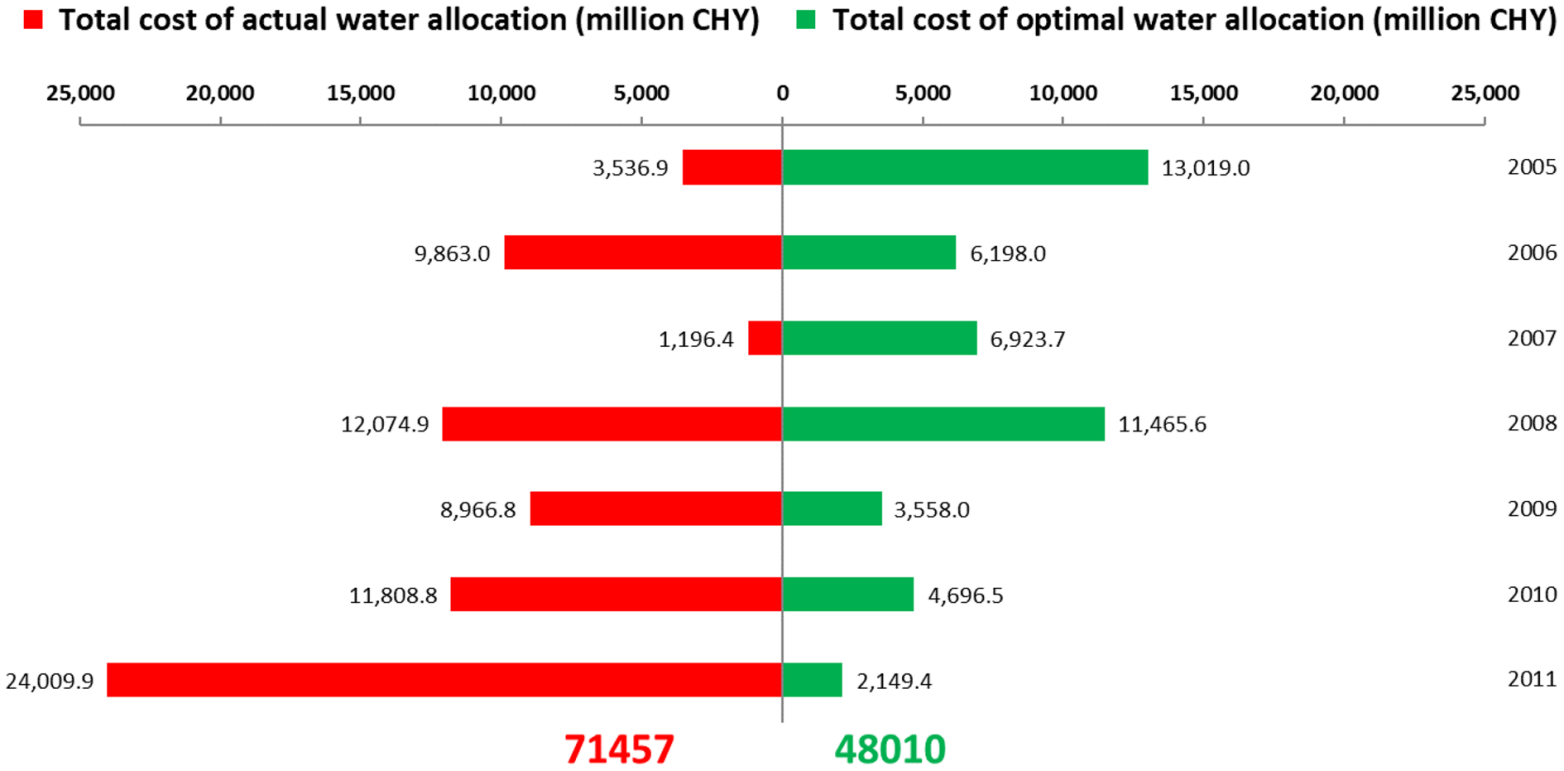
Comparison of the cost of actual and optimal water allocation.

The cost of the optimal allocated water was only 48 billion CHY, which is much less than 71.4 billion CHY in actual allocated water (Table S6; Fig. 5). In 2005 and 2007, the cost of optimal allocated water was higher than that of actual allocated water, and the cost of optimal allocated water decreased after 2008. From 2009 to 2011, the cost of optimal allocated water was significantly lower than that of actual allocated water. This stems from the fact that the water demand satisfied ratios in the actual water allocation scheme were all 100% from 2005 to 2008 and were higher than those under optimal water allocation (Table 2). This suggests that water demand was not fully met in the optimal allocation scheme, and the allocated water cost and opportunity loss for not meeting water demand in the optimal allocation scheme were greater than that of the actual allocation scheme, even if there was a loss of the penalty for exceeding water demand in the actual water allocation scheme. The unit loss of the opportunity loss is greater than the water price and the unit loss of the penalty loss for each water use sector (Table S1); the opportunity loss is much greater than both the allocated water cost and penalty loss, and it accounts for much of the total cost of water allocation, demonstrating that the total cost of the optimal water allocation scheme (i.e., with lower water demand satisfied ratio and higher opportunity loss) was greater than that of the actual water allocation scheme (i.e., with higher water demand satisfied ratio and lower opportunity loss). From 2009 to 2011, the water demand satisfied ratios in the optimal water allocation scheme were higher than those in the actual water allocation scheme, which decreases the opportunity loss and the total cost of water allocation across the three years.

From the perspective of uncertainty, the optimal allocation of water resources can be divided into deterministic allocation and uncertain allocation. In light of the uncertainty in available water and water demand, water allocation by the WAINM is classified as uncertain allocation. The water allocation results by the WAINM were compared with the water allocation results from the water resources allocation scheme in the Dongjiang River Basin of Guangdong Province, which was obtained via the deterministic allocation model using a multi-objective analysis method (i.e., MOA model) (Table S7). Compared with actual water use, water shortage occurred in both of the water allocation schemes obtained by the WAINM and MOA models (the WAINM allocated more water for agricultural water use, which resulted in abandoned water) (Table S7). The water shortage of the water allocation schemes obtained by the MOA model was much greater than that of the WAINM, especially water allocation for agricultural water use, as the MOA model applied the water supply design guarantee rate parameter when attempting to optimize allocation decisions^45^. In the MOA model, the water supply guarantee rate of agricultural water use was the lowest; the agricultural water shortage was the most severe. The water shortage of the industrial water allocation scheme obtained by the MOA model was less than that of the WAINM, which mainly stemmed from the higher guarantee rate of the industrial water supply^46^. Nevertheless, the total water shortage in the optimal water allocation scheme obtained by the MOA model was 32.6 million m^3^, which was much higher than that of the water allocation scheme obtained by the WAINM with a water shortage of 3.1 million m^3^. The price parameters in Table S1 were used to calculate the cost of the two types of water allocation schemes. The water allocation cost of the MOA model was much higher than that of the WAINM (Table S7). The water shortage was less severe and the costs were lower for the optimal water allocation scheme obtained by the WAINM under an uncertain water supply compared with the traditional deterministic MOA model; these results were also more similar to actual water use.

### Sensitivity analysis of the three cost price parameters in the water allocation improved newsvendor model

**Figure 6:**
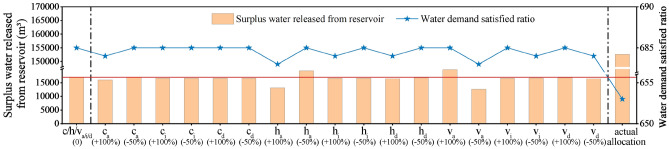
Changes in *c*, *h* and *v* on the optimal water allocation obtained in terms of surplus water released from reservoir and score of water demand satisfied ratio.

Figure 6 shows the impacts of changes in $c$, $h$ and $v$ on the optimal water allocation obtained by the WAINM in terms of surplus water released from reservoir and score. When the three parameters of each industry are doubled or decreased by 50%, both the surplus water released from reservoir and score of the corresponding optimal water allocation scheme show some changes, and the surplus water released from reservoir has greater change. In particular, the scores have barely changed, showing strong robustness of the WAINM. No matter how the three parameters change, the optimal water allocation shows obvious advantages over the actual water allocation, with less surplus water released from reservoir and higher guarantee rate of water supply.

Fig. S2 shows the change rates of optimal allocated water caused by doubled or decreased by 50% changes of $h$ and $v$ for each water use sector from 2005 to 2011, respectively. Obviously, in all years, changes of $c$ , $h$ and $v$ for agriculture lead to the largest change range of allocated water (i.e., with an average of 0.126% by $c$, 0.772% by $h$, and 0.887% by $v$), followed by industrial (i.e., with an average of 0.055% by $c$, 0.448% by $h$, and 0.500% by $v$), and domestic has the least change range (i.e., with an average of 0.016% by $c$, 0.149% by $h$, and 0.165% by $v$). Although the three parameters of agricultural water use were the lowest, the optimal allocated water caused by their changes were the biggest, because agricultural water use accounts for the most in the basin^47^. The optimal allocated water has the highest sensitivity to the change of $v$, followed by $h$, and the lowest sensitivity to the change of $c$, because $c$ is the lowest of the three parameters, and the change of $c$ has little effect on the allocation of water. The change of $h$ has greater impact, and the change of $v$ has the greatest impact, indicating that among the three price parameters of the WAINM, the opportunity loss has the greatest impact on the allocated water while the water price has the least impact.

The WAINM has several advantages compared with actual water allocation and the traditional deterministic MOA model. It helps decision makers determine the optimal water allocation schemes and achieve a stable water demand satisfied ratio for each water use sector at the lowest total cost; it also uses the CDFs to represent the uncertainties in both water demand and supply, which is more similar to the actual water resources system.

The optimal water allocation amount obtained by the WAINM for each water use sector is determined by the water price, the unit loss of the penalty for exceeding water demand, the unit loss of opportunity loss for not meeting the water demand of the water use sector, and the CDFs of water use and supply. The water price and the unit loss of the penalty and opportunity loss can be determined by the market or by the government; It is difficult to predict regional water demand, because the influencing factors are complex and dynamic, and water demand prediction is full of challenges with greater uncertainty^48,49^. In the current study, considering the short and stable time series of industrial water demand in the study area, meanwhile, the number of newspapers the newsvendor purchased every day in the Newsvendor model was assumed to conform to a uniform distribution^35–37^, the demand for newspapers is uncertain, just like the uncertainty in the water demand of each water use sector in the water resources allocation model, therefore, the uniform distribution was chosen to express the uncertainties in water demand. The parameter of the uniform distribution was calibrated and validated with time series of observed water use in the study area, and the uniform distribution for each water use sector was taken as the input for the WAINM.

### Practical applications and future research prospects

The novel proposed framework can be applied to provide optimal water allocation schemes for water governors in the fast-developing countries or regions with the strictest water resources management systems, according to the water price and the unit loss of the penalty and opportunity loss determined by the water market or by the government. The cumulative distributions applied in the WAINM can be adjusted in real time according to the water use characteristics of the region and the temporal distribution of reservoir inflows of the reservoir, and the impact of regional water demand and supply on the optimal water allocation amount should be further studied. Some constraints can be added in the WAINM to ensure the superiority of domestic water use in future studies.

## Conclusions

The current study proposed a newsvendor model-based framework for water allocation. The possibility distribution functions of both water demand and supply, as well as the cost of each water use sector, the unit loss of the exceeding water demand, and the unit loss of the penalty for not meeting water demand, were incorporated into the framework to determine the optimized water allocation schemes with lowest total costs. The case study suggests that, in the optimal water allocation scheme from the WAINM, the water demand satisfied ratio increased and the costs were lower compared with actual water allocation. The total water shortage and cost in the optimal water allocation scheme by the traditional deterministic MOA model were respectively 9.52 and 2.26 times larger than that of the water allocation scheme by the WAINM. Sensitivity analysis of the three cost price parameters demonstrated the strong robustness of the WAINM.

### Supplementary Information

Supplementary Information.

## Data Availability

Data will be available from the corresponding author on reasonable request.

## References

[1] Li N, Wang X, Shi M, Yang H (2015). Economic impacts of total water use control in the Heihe river basin in Northwestern China—An integrated CGE-BEM modeling approach. Sustainability.

[2] Zhou F (2020). Deceleration of China’s human water use and its key drivers. Proc. Natl. Acad. Sci..

[3] Giuliani M, Lamontagne JR, Reed PM, Castelletti A (2021). A state-of-the-art review of optimal reservoir control for managing conflicting demands in a changing world. Water Resour. Res..

[4] Steinfeld CM, Sharma A, Mehrotra R, Kingsford RT (2020). The human dimension of water availability: Influence of management rules on water supply for irrigated agriculture and the environment. J. Hydrol..

[5] Macian-Sorribes H, Pulido-Velazquez M (2020). Inferring efficient operating rules in multireservoir water resource systems: A review. Wiley Interdiscip. Rev. Water.

[6] Rani D, Moreira MM (2010). Simulation-optimization modeling: A survey and potential application in reservoir systems operation. Water Resour. Manag..

[7] Li M (2020). Efficient irrigation water allocation and its impact on agricultural sustainability and water scarcity under uncertainty. J. Hydrol..

[8] Nguyen-Ky T (2018). Predicting water allocation trade prices using a hybrid Artificial Neural Network-Bayesian modelling approach. J. Hydrol..

[9] Zeng Y, Li J, Cai Y, Tan Q, Dai C (2019). A hybrid game theory and mathematical programming model for solving trans-boundary water conflicts. J. Hydrol..

[10] Liu D (2018). Assessing the effects of adaptation measures on optimal water resources allocation under varied water availability conditions. J. Hydrol..

[11] Du E, Cai X, Wu F, Foster T, Zheng C (2021). Exploring the impacts of the inequality of water permit allocation and farmers’ behaviors on the performance of an agricultural water market. J. Hydrol..

[12] Harou JJ (2009). Hydro-economic models: Concepts, design, applications, and future prospects. J. Hydrol..

[13] Maier HR (2014). Evolutionary algorithms and other metaheuristics in water resources: Current status, research challenges and future directions. Environ. Modell. Softw..

[14] ReVelle C (2000). Research challenges in environmental management. Eur. J. Oper. Res..

[15] Tian Y, Chi H, Li H, Shan J, Zhai C (2005). Research on mathematical simulation of residual chlorine decay and optimization of chlorination allocation of urban water distribution system. J. Hydrodyn..

[16] Tabari M, Soltani J (2013). Multi-objective optimal model for conjunctive use management using SGAs and NSGA-II models. Water Resour. Manage.

[17] Leidner A, Rister M, Lacewell R, Woodard J, Sturdivant A (2012). An analysis of input choice, input prices, and environmental factors on the costs of seawater reverse osmosis systems. Desalination.

[18] Maiolo M, Pantusa D (2016). An optimization procedure for the sustainable management of water resources. Water Supply..

[19] Yang X (2022). Decision optimization for water and electricity shared resources based on fusion swarm intelligence. Axioms..

[20] McClanahan B (2014). Green and grey: Water justice, criminalization, and resistance. Crit. Criminol..

[21] Johnson E, Schwartz J, Inlow A (2020). The criminalization of environmental harm: a study of the most serious environmental offenses prosecuted by the US federal government, 1985–2010. Environ. Sociol..

[22] Wang B (2015). Urban water resources allocation under the uncertainties of water supply and demand: a case study of Urumqi, China. Environ. Earth Sci..

[23] Sheibani H, Alizadeh H, Shourian M (2019). Optimum design and operation of a reservoir and irrigation network considering uncertainty of hydrologic, agronomic and economic factors. Water Resour. Manag..

[24] Wei F, Zhang X, Xu J, Bing J, Pan G (2020). Simulation of water resource allocation for sustainable urban development: An integrated optimization approach. J. Cleaner Prod..

[25] He Y, Gong Z, Zheng Y, Bai X, Wang P (2021). The water dispatching of river basins during dry periods under the most stringent water management system in China. Water Policy.

[26] Zhao Y, Shen Y, Yan J (2021). Design and application of genetic algorithm based on signal game and newsboy model for optimizing supply chain. Discrete Dyn. Nat. Soc..

[27] Qin Y, Wang R, Vakharia AJ, Chen Y, Şeref MM (2011). The newsvendor problem: Review and directions for future research. Eur. J. Oper. Res..

[28] Zhang LL, Yang Y, Cai JQ (2020). One-way substitution newsboy problem under retailer’s budget constraint. Math. Probl. Eng..

[29] Wu D, Chen F (2020). The overconfident and ambiguity-averse newsvendor problem in perishable product decision. Comput. Indust. Eng..

[30] Wang F, Wu D, Yu H, Shen H, Zhao Y (2021). Understanding the role of big data analytics for coordination of electronic retail service supply chain. J. Enterp. Inf. Manag..

[31] Chan FT, Xu X (2019). The loss-averse retailer’s order decisions under risk management. Mathematics.

[32] Wan Z, Zhu S, Wan Z (2020). An integrated stochastic model and algorithm for multi-product newsvendor problems. Inter. J. Model. Simul. Sci. Comput..

[33] Chou S, Chen CW (2017). Supply chain coordination: an inventory model for single-period utility product under fuzzy demand. Int. J. Adv. Manuf. Technol..

[34] Lotfi R, Weber GW, Sajadifar SM, Mardani N (2020). Interdependent demand in the two-period newsvendor problem. J. Indust. Manag. Optim..

[35] Wang T, Hu Q (2013). Risk-averse newsvendor model with strategic consumer behavior. J. Appl. Math..

[36] Pando V, San-Jose L, Garcia-Laguna J, Sicilia J (2014). A newsvendor inventory model with an emergency order to supply a non-increasing fraction of shortage. Appl. Math. Comput..

[37] Guler M (2022). An analysis on the marketing budget of a newsvendor. Appl. Math. Comput..

[38] He Y, Chen X, Sheng Z, Lin K, Gui F (2018). Water allocation under the constraint of total water-use quota: a case from Dongjiang River Basin, South China. Hydrol. Sci. J..

[39] Faria LC (2019). Irrigation distribution uniformity analysis on a lateral-move irrigation system. Irrig. Sci..

[40] Samadi S, Pourreza-Bilondi M, Wilson CAME, Hitchcock DB (2020). Bayesian model averaging with fixed and flexible priors: Theory, concepts, and calibration experiments for rainfall-runoff modeling. J. Adv. Model. Earth Syst..

[41] Silva AS, Ghisi E (2016). Uncertainty analysis of daily potable water demand on the performance evaluation of rainwater harvesting systems in residential buildings. J. Environ. Manag..

[42] Reis J, Shortridge J (2020). Impact of uncertainty parameter distribution on robust decision making outcomes for climate change adaptation under deep uncertainty. Risk Anal..

[43] Liu Z, Yang H, Wang T (2021). A simple framework for estimating the annual runoff frequency distribution under a non-stationarity condition. J. Hydrol..

[44] Yan Y, Ju H, Zhang S, Jiang W (2020). Spatiotemporal patterns and driving forces of urban expansion in coastal areas: A study on urban agglomeration in the pearl river delta, China. Sustainability.

[45] Li J, Song S, Ayantobo O, Wang H, Jiaping L, Zhang B (2022). Coordinated allocation of conventional and unconventional water resources considering uncertainty and different stakeholders. J. Hydrol..

[46] Liu D (2012). A macro-evolutionary multi-objective immune algorithm with application to optimal allocation of water resources in Dongjiang River basins, South China. Stoch. Environ. Res. Risk Assess..

[47] Zhou Z (2023). Bivariate socioeconomic drought assessment based on a hybrid framework and impact of human activities. J. Clean. Prod..

[48] Zhang Q, Diao Y, Dong J (2013). Regional water demand prediction and analysis based on Cobb-Douglas model. Water Resour. Manag..

[49] Yang Z (2023). Water consumption prediction and influencing factor analysis based on PCA-BP neural network in karst regions: a case study of Guizhou Province. Environ. Sci. Pollut. Res..

