# Supplementary material for: Water resources allocation considering water supply and demand uncertainties using newsvendor model-based framework
Source: Sci Rep. 2023 Aug 22;13:13639. doi: 10.1038/s41598-023-40692-7 (PMC10444857; doi:10.1038/s41598-023-40692-7)
Supplement: Supplementary file 1 — Supplementary Information. [file 41598_2023_40692_MOESM1_ESM.docx]

**Water Resources Allocation Considering Water Supply and Demand Uncertainties Using Newsvendor Model-based Framework**

Yanhu He^1,2^, Yanhui Zheng^3,4*^, Xiaohong Chen^5^, Binfen Liu^1^, Qian Tan^1^

**D1: The derivation procedure of the optimal allocated water (**${\boldsymbol{x}_{\boldsymbol{i,j}}^{\boldsymbol{t}}}^{\boldsymbol{*}}$**) without considering the constraint of reservoir inflow**

The expected cost function of water allocation of $j_{th}$ water use sector in the $i_{th}$ calculation unit is as follow:

$E\left[ C\left( x \right) \right]=E[C\left( x_{i,j}^{t} \right)]={E[C}_{o}\left( x_{i,j}^{t} \right)+C_{u}\left( x_{i,j}^{t} \right)+C_{p}(x_{i,j}^{t})]$

The expected cost functions of $C_{o}\left( x_{i,j}^{t} \right),$ $C_{u}\left( x_{i,j}^{t} \right)$ and $C_{p}\left( x_{i,j}^{t} \right)$ are as follows:

$E[C_{o}\left( x_{i,j}^{t} \right)]=h\cdot\int_{0}^{\infty} g\left( R^{t} \right)\int_{0}^{x_{i,j}^{t}} \left( x_{i,j}^{t}-D_{i,j}^{t} \right)\cdot f_{i,j}\left( D_{i,j}^{t} \right)\cdot dD_{i,j}^{t}\cdot dR^{t}$

$E[C_{u}\left( x_{i,j}^{t} \right)]=v\cdot\int_{0}^{\infty} g\left( R^{t} \right)\int_{x_{i,j}^{t}}^{\infty} \left( D_{i,j}^{t}-x_{i,j}^{t} \right)\cdot f_{i,j}\left( D_{i,j} \right)\cdot dD_{i,j}^{t}\cdot dR^{t}$

$E[C_{p}\left( x_{i,j}^{t} \right)]=c\cdot x_{i,j}^{t}$

Thus, the first derivative of $E\left[ C\left( x \right) \right]$ is as follow:

$\frac{\partial E[C\left( x_{i,j}^{t} \right)]}{\partial x_{i,j}^{t}}=\left( h+v \right)F_{i,j}\left( x_{i,j}^{t} \right)-1+c$

The second derivative of $E\left[ C\left( x \right) \right]$ is as follow:

$\frac{\partial^{2}E[C\left( x_{i,j}^{t} \right)]}{\partial x_{i,j}^{t}}=\left( h+v \right)f_{i,j}\left( x_{i,j}^{t} \right)>0$

Therefore, $C\left( x_{i,j}^{t} \right)$ is a concave function, and it needs to be minimized to obtain the optimal allocated water. The upper and lower bounds of runoff and water demand should be compared to define $C\left( x_{i,j}^{t} \right)$.

$D_{i,jmin}^{t}<D_{i,j}^{t}<D_{i,jmax}^{t}$

$R_{min}^{t}<R^{t}<R_{max}^{t}$

If $R_{max}^{t}<D_{i,jmax}^{t}$,

$E[C_{o}\left( x_{i,j}^{t} \right)]=h\cdot\int_{R_{min}^{t}}^{R_{max}^{t}} g\left( R \right)\int_{0}^{x_{i,j}^{t}} \left( x_{i,j}^{t}-D_{i,j}^{t} \right)\cdot f_{i,j}\left( D_{i,j}^{t} \right)\cdot dD_{i,j}^{t}\cdot dR^{t}$

$E[C_{u}\left( x_{i,j}^{t} \right)]=v\cdot\int_{R_{min}^{t}}^{R_{max}^{t}} g\left( R \right)\int_{x_{i,j}^{t}}^{D_{i,jmax}^{t}} \left( D_{i,j}^{t}-x_{i,j}^{t} \right)\cdot f_{i,j}\left( D_{i,j}^{t} \right)\cdot dD_{i,j}^{t}\cdot dR^{t}$

$\frac{\partial E[C_{o}\left( x_{i,j}^{t} \right)]}{\partial x_{i,j}^{t}}=hF_{i,j}\left( x_{i,j}^{t} \right)$

$\frac{\partial E[C_{u}\left( x_{i,j}^{t} \right)]}{\partial x_{i,j}^{t}}=v\left( F_{i,j}\left( x_{i,j}^{t} \right)-1 \right)$

$\frac{\partial E[C\left( x_{i,j}^{t} \right)]}{\partial x_{i,j}^{t}}=\left( h+v \right)F_{i,j}\left( x_{i,j}^{t} \right)-v+c$

when $\frac{\partial E[C\left( x_{i,j}^{t} \right)]}{\partial x_{i,j}^{t}}=0$,

$$F_{i,j}\left( {x_{i,j}^{t}}^{*} \right)=\frac{v-c}{h+v}$$

If $R_{max}^{t}>D_{i,jmax}^{t}$,

$E[C_{o}\left( x_{i,j}^{t} \right)]=h\cdot\int_{R_{min}^{t}}^{D_{i,jmax}^{t}} g\left( R^{t} \right)\int_{0}^{x_{i,j}^{t}} \left( x_{i,j}^{t}-D_{i,j}^{t} \right)\cdot f_{i,j}\left( D_{i,j}^{t} \right)\cdot dD_{i,j}^{t}\cdot dR^{t}+h\cdot\int_{D_{i,jmax}^{t}}^{R_{max}^{t}} g\left( R^{t} \right)\int_{0}^{D_{i,jmax}^{t}} \left( x_{i,j}^{t}-D_{i,j}^{t} \right)\cdot f_{i,j}\left( D_{i,j}^{t} \right)\cdot dD_{i,j}^{t}\cdot dR^{t}$

$E[C_{u}\left( x_{i,j}^{t} \right)]=v\cdot\int_{R_{min}^{t}}^{D_{i,jmax}^{t}} g\left( R^{t} \right)\int_{x_{i,j}^{t}}^{D_{i,jmax}^{t}} \left( D_{i,j}^{t}-x_{i,j}^{t} \right)\cdot f_{i,j}\left( D_{i,j}^{t} \right)\cdot dD_{i,j}^{t}\cdot dR^{t}$

when $\frac{\partial E[C\left( x_{i,j}^{t} \right)]}{\partial x_{i,j}^{t}}=0$,

$F_{i,j}\left( {x_{i,j}^{t}}^{*} \right)=1-\frac{h+c}{(h+v)G(D_{i,jmax}^{t})}$

${x_{i,j}^{t}}^{*}$ is the optimal allocated water, $F_{i,j}()$ represents the cumulative distribution function (CDF) of the water demand.

**D2: The derivation procedure of the optimal allocated water (**${\boldsymbol{x}_{\boldsymbol{i,j}}^{\boldsymbol{t}}}^{\boldsymbol{*}}$**) without considering the constraint of reservoir inflow**

The new Lagrange function is as follows:

$L(x_{i,j}^{t},\lambda)=\sum_{i=1}^{I} \sum_{j=1}^{J} [C_{o}\left( x_{i,j}^{t} \right)+C_{u}\left( x_{i,j}^{t} \right)+C_{p}\left( x_{i,j}^{t} \right)]+\lambda(\sum_{i=1}^{I} \sum_{j=1}^{J} x_{i,j}^{t}-Q_{max}^{t})$

$\lambda$ represents the Lagrange parameter.

If $l\left( x_{i,j}^{t},\lambda\right)=C_{o}\left( x_{i,j}^{t} \right)+C_{u}\left( x_{i,j}^{t} \right)+C_{p}\left( x_{i,j}^{t} \right)+\lambda(x_{i,j}^{t}-Q_{max}^{t})$

The following equation can be obtained according to the rule of taking the derivative of the sum of functions:

$$L^{’}\left( x_{1,1},x_{1,2},\ldots,x_{i,j}^{t},\lambda\right)=l^{’}\left( x_{i,j}^{1},\lambda\right)+l^{’}\left( x_{i,j}^{2},\lambda\right)\ldots+l^{’}\left( x_{i,j}^{T},\lambda\right)=\sum_{t=1}^{T} l^{'}\left( x_{i,j}^{t},\lambda\right)$$

The optimal allocated water ${x_{i,j}^{t}}^{*}$ can be obtained as follow:

If $R_{max}<D_{i,jmax}$, when $\frac{\partial l\left( x_{i,j}^{t},\lambda\right)}{\partial x_{i,j}^{t}}=0$, $F_{i,j}\left( {x_{i,j}^{t}}^{*} \right)=\frac{v-c-\lambda}{h+v}$

If $R_{max}>D_{i,jmax}$,

$$C_{o}\left( x_{i,j}^{t} \right)=h\cdot\int_{R_{min}^{t}}^{D_{i,jmax}^{t}} g\left( R^{t} \right)\int_{0}^{x_{i,j}^{t}} \left( x_{i,j}^{t}-D_{i,j}^{t} \right)\cdot f_{i,j}\left( D_{i,j}^{t} \right)\cdot dD_{i,j}^{t}\cdot dR^{t}+h\cdot\int_{D_{i,jmax}^{t}}^{R_{max}^{t}} g\left( R^{t} \right)\int_{0}^{D_{i,jmax}^{t}} \left( x_{i,j}^{t}-D_{i,j}^{t} \right)\cdot f_{i,j}\left( D_{i,j}^{t} \right)\cdot dD_{i,j}^{t}\cdot dR^{t}$$

$C_{u}\left( x_{i,j}^{t} \right)=v\cdot\int_{R_{min}^{t}}^{D_{i,jmax}^{t}} g\left( R^{t} \right)\int_{x_{i,j}^{t}}^{D_{i,jmax}^{t}} \left( D_{i,j}^{t}-x_{i,j}^{t} \right)\cdot f_{i,j}\left( D_{i,j}^{t} \right)\cdot dD_{i,j}^{t}\cdot dR^{t}$

when $\frac{\partial l\left( x_{i,j}^{t},\lambda\right)}{\partial x_{i,j}^{t}}=0$, $F_{i,j}\left( {x_{i,j}^{t}}^{*} \right)=1-\frac{h+c-\lambda}{(h+v)G(D_{i,jmax}^{t})}$

where ${x_{i,j}^{t}}^{*}$ is the optimal allocated water and it can be represented by $\lambda$. An optimal value of $\lambda$ is calculated to obtain the optimal allocated water${x_{i,j}^{t}}^{*}$.

Figure caption

Fig. S1 The frequency curves of the monthly inflow of the Baipenzhu reservoir

Fig. S2 Change rates of optimal allocated water caused by doubled or decreased by 50% changes in c, h and v for each water use sector


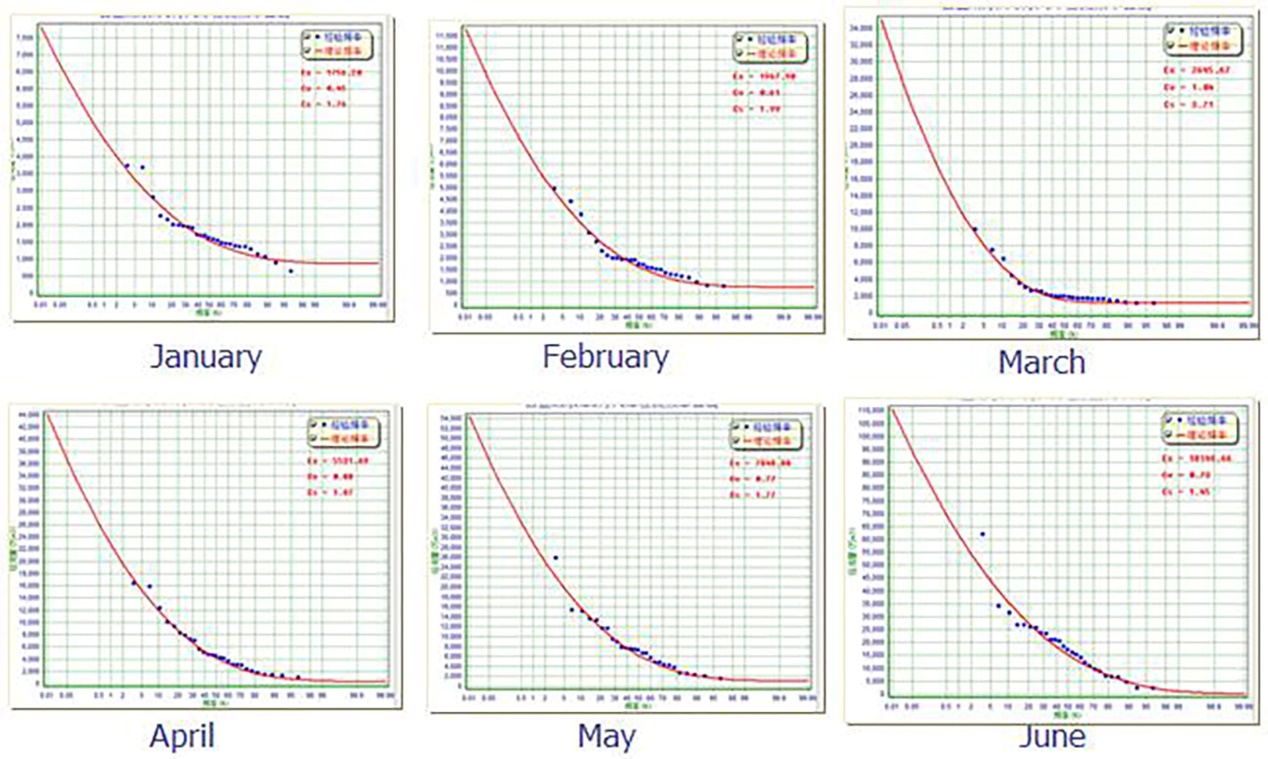


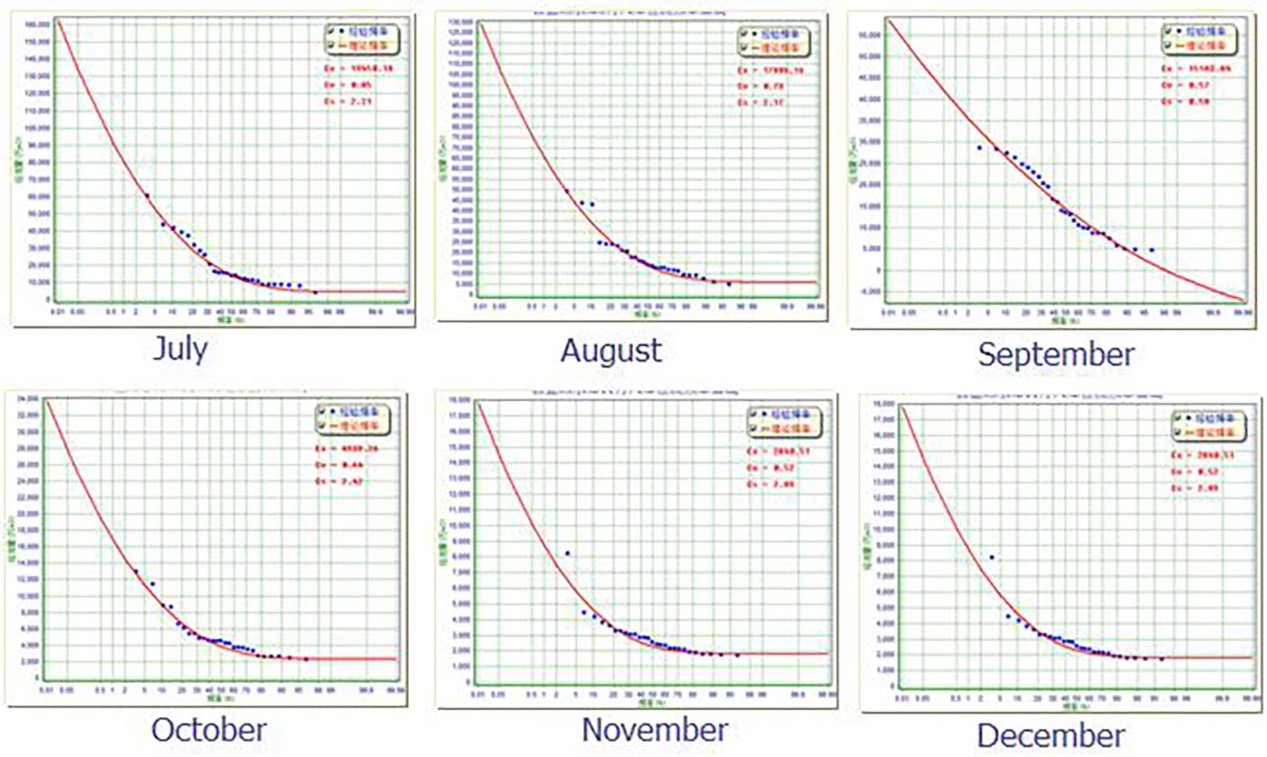


Fig. S1 The frequency curves of the monthly inflow of the Baipenzhu reservoir(this figure was generated using Delphi 7.0. (https://www.embarcadero.com/products/delphi/starter)


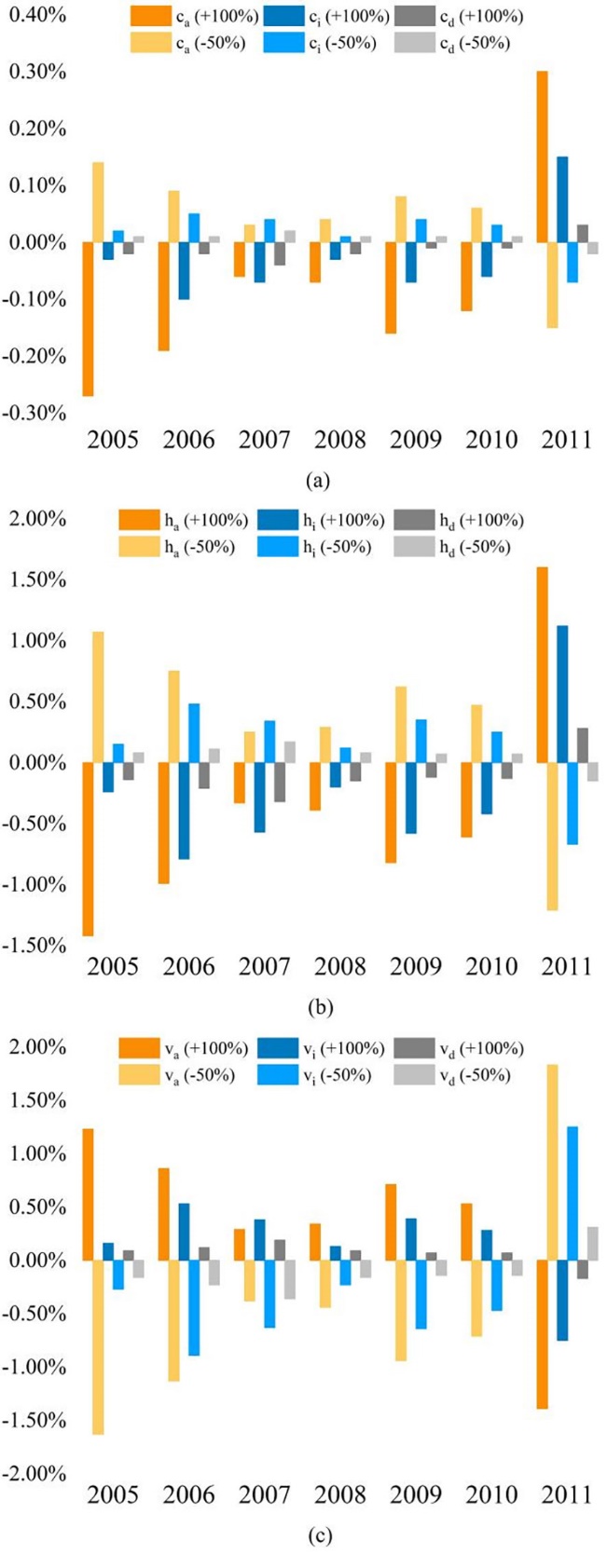


Fig. S2 Change rates of optimal allocated water caused by doubled or decreased by 50% changes in c, h and v for each water use sector

Table captions

Table. S1 Water price, the unit loss of the penalty and opportunity loss

Table. S2 The correlation coefficient of monthly reservoir inflow fitting the frequency curves

Table. S3 The intra-annual distribution coefficients of each water use sector

Table. S4 Actual allocated water from 2005 to 2011 (million m^3^)

Table. S5 Comparison of the water surplus for each industry under actual and optimal water allocation (million m^3^)

Table. S6 Comparison of the total cost of actual and optimal allocated water for each industry (million CHY)

Table. S7 Comparison of the water allocation results from the Water allocation based on the Improved Newsvendor Model and Multi-Objective Analysis model

Table. S1 Water price, the unit loss of the penalty and opportunity loss (m^3^/s)

| Item | Huidong | Huiyang |
| --- | --- | --- |
| *c_a_* | 0.5 | 0.5 |
| *c_i_* | 2.38 | 3.18 |
| *c_d_* | 2.765 | 2.95 |
| *h_a_* | 5 | 5 |
| *h_i_* | 23.8 | 31.8 |
| *h_d_* | 27.65 | 29.5 |
| *v_a_* | 12 | 12 |
| *v_i_* | 222 | 148 |
| *v_d_* | 351 | 234 |

Note: *c_a,_ c_i_* and *c_d_* respectively represent water price of agriculture, industrial and domestic; *h_a,_ h_i_* and *h_d_* represent the unit loss of the penalty; *v_a,_ v_i_* and *v_d_* represents the unit loss of the opportunity loss.

Table. S2 The correlation coefficient of monthly reservoir inflow fitting the frequency curves

| Month | Correlation coefficient | Month | Correlation coefficient |
| --- | --- | --- | --- |
| Jan | 0.93 | Jul | 0.97 |
| Feb | 0.95 | Aug | 0.95 |
| Mar | 0.97 | Sep | 0.96 |
| Apr | 0.98 | Oct | 0.97 |
| May | 0.96 | Nov | 0.9 |
| Jun | 0.92 | Dec | 0.98 |

Table. S3 The intra-annual distribution coefficients of each water use sector

| Industry | Month | | | | | | | | | | | |
| --- | --- | --- | --- | --- | --- | --- | --- | --- | --- | --- | --- | --- |
|  | Jan | Feb | Mar | Apr | May | Jun | Jul | Aug | Sep | Oct | Nov | Dec |
| Agriculture | 0.026 | 0.026 | 0.026 | 0.053 | 0.105 | 0.211 | 0.184 | 0.158 | 0.105 | 0.053 | 0.026 | 0.026 |
| Industrial | 0.083 | 0.083 | 0.083 | 0.083 | 0.083 | 0.083 | 0.083 | 0.083 | 0.083 | 0.083 | 0.083 | 0.083 |
| Domestic | 0.083 | 0.083 | 0.083 | 0.083 | 0.083 | 0.083 | 0.083 | 0.083 | 0.083 | 0.083 | 0.083 | 0.083 |

Table. S4 Actual allocated water from 2005 to 2011 (million m^3^)

| Year | Huidong | | | Huiyang | | | The whole basin | | | |
| --- | --- | --- | --- | --- | --- | --- | --- | --- | --- | --- |
|  | Agricultural | Industrial | Domestic | Agricultural | Industrial | Domestic | Agricultural | Industrial | Domestic | Total |
| 2005 | 465.3 | 139.6 | 106.4 | 166.3 | 49.9 | 38.0 | 631.7 | 189.5 | 144.4 | 965.5 |
| 2006 | 669.6 | 198.2 | 170.6 | 239.4 | 70.9 | 61.0 | 908.9 | 269.1 | 231.5 | 1409.5 |
| 2007 | 368.0 | 123.0 | 98.5 | 131.6 | 44.0 | 35.2 | 499.6 | 167.0 | 133.8 | 800.4 |
| 2008 | 641.4 | 270.4 | 195.3 | 229.3 | 96.6 | 69.8 | 870.7 | 367.0 | 265.2 | 1502.8 |
| 2009 | 313.4 | 133.3 | 85.7 | 112.0 | 47.6 | 30.6 | 425.5 | 180.9 | 116.3 | 722.7 |
| 2010 | 291.7 | 136.7 | 87.9 | 104.3 | 48.9 | 31.4 | 395.9 | 185.6 | 119.3 | 700.8 |
| 2011 | 248.6 | 112.6 | 74.6 | 88.9 | 40.3 | 26.7 | 337.5 | 152.9 | 101.3 | 591.6 |

Table. S5 Comparison of the water surplus for each industry under actual and optimal water allocation (million m^3^)

| Year | | | 2005 | 2006 | 2007 | 2008 | 2009 | 2010 | 2011 |
| --- | --- | --- | --- | --- | --- | --- | --- | --- | --- |
| Actual water allocation | Huidong | Agriculture | 89.2 | 299.3 | 4.8 | 299.1 | 0.0 | 0.0 | 0.0 |
|  |  | Industrial | 26.7 | 88.6 | 1.6 | 126.1 | 0.0 | 0.0 | 0.0 |
|  |  | Domestic | 20.4 | 76.2 | 1.3 | 91.1 | 0.0 | 0.0 | 0.0 |
|  | Huiyang | Agriculture | 31.9 | 107.0 | 1.7 | 106.9 | 0.0 | 0.0 | 0.0 |
|  |  | Industrial | 9.6 | 31.7 | 0.6 | 45.1 | 0.0 | 0.0 | 0.0 |
|  |  | Domestic | 7.3 | 27.3 | 0.5 | 32.6 | 0.0 | 0.0 | 0.0 |
|  | The whole basin | Agriculture | 121.0 | 406.3 | 6.5 | 406.0 | 0.0 | 0.0 | 0.0 |
|  |  | Industrial | 36.3 | 120.3 | 2.2 | 171.1 | 0.0 | 0.0 | 0.0 |
|  |  | Domestic | 27.7 | 103.5 | 1.7 | 123.7 | 0.0 | 0.0 | 0.0 |
|  |  | Total | **1526.3** | | | | | | |
| Optimal water allocation | Huidong | Agriculture | 16.5 | 15.0 | 8.8 | 28.9 | 14.5 | 24.4 | 0.0 |
|  |  | Industrial | 0.0 | 0.0 | 0.0 | 0.0 | 0.0 | 0.0 | 0.8 |
|  |  | Domestic | 0.0 | 0.0 | 0.0 | 0.0 | 9.1 | 3.7 | 2.2 |
|  | Huiyang | Agriculture | 6.8 | 6.0 | 3.3 | 10.6 | 5.7 | 9.1 | 0.0 |
|  |  | Industrial | 0.0 | 0.0 | 0.0 | 0.0 | 0.0 | 0.0 | 0.0 |
|  |  | Domestic | 0.0 | 0.0 | 0.0 | 0.0 | 3.1 | 1.2 | 0.5 |
|  | The whole basin | Agriculture | 23.3 | 21.0 | 12.1 | 39.5 | 20.2 | 33.6 | 0.0 |
|  |  | Industrial | 0.0 | 0.0 | 0.0 | 0.0 | 0.0 | 0.0 | 0.6 |
|  |  | Domestic | 0.0 | 0.0 | 0.0 | 0.0 | 12.2 | 4.9 | 2.7 |
|  |  | Total | **169.9** | | | | | | |

Table. S6 Comparison of the total cost of actual and optimal allocated water for each industry (million CHY)

| Year | | | 2005 | 2006 | 2007 | 2008 | 2009 | 2010 | 2011 |
| --- | --- | --- | --- | --- | --- | --- | --- | --- | --- |
| Actual  allocated water | Huidong | Agriculture | 678.4 | 1831.3 | 207.9 | 1816.1 | 535.7 | 614.9 | 1149.0 |
|  |  | Industrial | 968.8 | 2580.9 | 330.9 | 3643.8 | 3298.3 | 4393.0 | 8853.9 |
|  |  | Domestic | 857.5 | 2579.5 | 307.8 | 3058.7 | 3266.9 | 4378.2 | 9200.1 |
|  | Huiyang | Agriculture | 242.5 | 654.7 | 74.3 | 649.2 | 191.5 | 219.8 | 410.7 |
|  |  | Industrial | 462.7 | 1232.8 | 158.0 | 1740.4 | 862.0 | 1124.8 | 2174.2 |
|  |  | Domestic | 327.1 | 983.8 | 117.4 | 1166.6 | 812.5 | 1078.2 | 2222.1 |
|  | The whole basin | Agriculture | 920.9 | 2486.0 | 282.2 | 2465.4 | 727.1 | 834.7 | 1559.7 |
|  |  | Industrial | 1431.5 | 3813.7 | 488.9 | 5384.3 | 4160.3 | 5517.7 | 11028.1 |
|  |  | Domestic | 1184.5 | 3563.4 | 425.3 | 4225.3 | 4079.4 | 5456.3 | 11422.1 |
|  |  | Total | 3536.9 | 9863.0 | 1196.4 | 12074.9 | 8966.8 | 11808.8 | 24009.9 |
| Optimal  allocated water | Huidong | Agriculture | 279.0 | 267.6 | 229.8 | 330.0 | 252.2 | 299.8 | 838.1 |
|  |  | Industrial | 7717.9 | 804.8 | 3061.1 | 5683.2 | 1830.3 | 2890.5 | 381.1 |
|  |  | Domestic | 2355.5 | 3650.2 | 2039.7 | 3063.2 | 536.8 | 388.2 | 344.6 |
|  | Huiyang | Agriculture | 104.4 | 99.0 | 83.3 | 119.3 | 93.1 | 109.4 | 263.2 |
|  |  | Industrial | 1934.4 | 423.2 | 911.7 | 1466.8 | 645.2 | 864.8 | 200.4 |
|  |  | Domestic | 627.9 | 953.2 | 598.1 | 803.0 | 200.4 | 143.8 | 122.0 |
|  | The whole basin | Agriculture | 383.3 | 366.6 | 313.1 | 449.4 | 345.3 | 409.2 | 1101.2 |
|  |  | Industrial | 9652.3 | 1227.9 | 3972.8 | 7150.0 | 2475.4 | 3755.3 | 581.5 |
|  |  | Domestic | 2983.4 | 4603.4 | 2637.8 | 3866.2 | 737.2 | 532.0 | 466.6 |
|  |  | Total | 13019.1 | 6198.0 | 6923.7 | 11465.6 | 3558.0 | 4696.5 | 2149.4 |

Table. S7 Comparison of the water allocation results from the Water allocation based on the Improved Newsvendor Model and Multi-Objective Analysis model

| Water use sector | Actual water use (million m³) | Allocated water (million m³) | | Water deficit （million m³） | | Water surplus （million m³） | | Total cost （million CHY） | |
| --- | --- | --- | --- | --- | --- | --- | --- | --- | --- |
|  |  | WAINM | MOA | WAINM | MOA | WAINM | MOA | WAINM | MOA |
| Agriculture | 510.6 | 533.9 | 282.9 | 0.0 | 227.7 | 23.3 | 0.0 | 383.3 | 2874.1 |
| Industrial | 153.2 | 106.8 | 141.9 | 46.4 | 11.3 | 0.0 | 0.0 | 9652.3 | 11466.5 |
| Domestic | 116.7 | 108.3 | 29.3 | 8.4 | 87.4 | 0.0 | 0.0 | 2983.4 | 28148.8 |
| Total | 780.5 | 749.0 | 454.1 | 54.8 | 326.4 | 0.0 | 0.0 | 13019.0 | 42489.4 |
